# Spatial Clustering of Receptors and Signaling Molecules Regulates NK Cell Response to Peptide Repertoire Changes

**DOI:** 10.3389/fimmu.2019.00605

**Published:** 2019-04-05

**Authors:** Berenice Mbiribindi, Sayak Mukherjee, Dannielle Wellington, Jayajit Das, Salim I. Khakoo

**Affiliations:** ^1^Department of Clinical and Experimental Sciences, Faculty of Medicine, University of Southampton, Southampton, United Kingdom; ^2^Institute of Bioinformatics and Applied Biotechnology, Bangalore, India; ^3^Department of Cancer Sciences, Faculty of Medicine, University of Southampton, Southampton, United Kingdom; ^4^Battelle Center for Mathematical Medicine, The Research Institute at the Nationwide Children's Hospital, Columbus, OH, United States; ^5^Department of Pediatrics, Wexner College of Medicine, The Ohio State University, Columbus, OH, United States; ^6^Biophysics Program, The Ohio State University, Columbus, OH, United States

**Keywords:** NK cells, peptide antagonism, KIR, HLA-C, signaling, modeling

## Abstract

Natural Killer (NK) cell activation requires integration of inhibitory and activating signaling. Inhibitory signals are determined by members of the killer cell immunoglobulin-like receptor (KIR) family, which have major histocompatibility complex (MHC) class I ligands. Loss of this inhibitory signal leads to NK cell activation. Thus, down-regulation of MHC I during viral infection or cancer induces NK cell activation. However, NK cell activation in the presence of MHC-I has been demonstrated for HLA-C^*^0102 through changes in its peptide content: “peptide antagonism.” Here we identify an antagonist peptide for HLA-C^*^0304 suggesting that peptide antagonism is a generalizable phenomenon and, using a combination of mathematical modeling, confocal imaging, and immune-assays, we quantitatively determine mechanisms that underlie peptide antagonism in inhibitory KIR2DL2/3 signaling. These data provide a mechanism for NK cell activation based on a reduction of inhibitory signaling in the presence of preserved levels of MHC class I.

## Introduction

Natural Killer (NK) cells play an important role in protection against viruses and tumor growth. Their response requires integration of inhibitory and activating signals. Inhibitory signals are determined by members of the killer cell immunoglobulin-like receptor (KIR) family, which bind to MHC class I ligands. The classical model by which NK cells lose tonic inhibitory signaling is well-described by the “missing-self hypothesis” (1, 2) such that down-regulation of MHC I during viral infection or tumorigenesis can induce NK cell activation (3–5). In addition to MHC class I down-regulation, viral infection and tumorigenesis can also induce changes in the peptide content of MHC class I. For tumors this includes expression of mutated proteins or up-regulation of tumor antigens, but in the early stages of viral infection there can be dramatic changes in the levels of presentation of viral peptides (6). KIR binding to MHC class I is determined both by the heavy chain of MHC class I and by its bound peptide (7). Peptides that engage KIR have specific motifs at the C terminal residues. For instance, in general, KIR2DL2 and KIR2DL3 (KIR2DL2/3) both recognize HLA-C molecules with asparagine at residue 80 of the MHC class I heavy chain (group 1 HLA-C) in combination with peptides which have a large hydrophobic amino acid at the C-terminal –2 position and a small amino acid at the C terminal-1 position (8–10). Additionally, the influence of peptide on KIR engagement can induce binding of KIR2DL2/3 to group 2 HLA-C molecules which have lysine at position 80 of the MHC class I heavy chain (11).

We have previously investigated the functional consequences of this peptide selectivity of KIR. We studied the group 1 HLA-C allele, HLA-C^*^0102, and identified peptides that did not induce inhibition of KIR2DL2/3-positive NK cells in isolation, but were able to disrupt the inhibition due to strong inhibitory peptides (12). We termed these antagonist peptides, as only a relatively small quantity of an antagonist peptide was required to disrupt KIR signaling. We also showed that these peptides were able to induce diffuse clustering of KIR at the immune synapse, and recruitment of SHP-1 to the inhibitory KIR, but this was not sufficient to dephosphorylate Vav1 (13, 14). This phenomenon provides an alternative to the “missing-self” model for losing an inhibitory signal from KIR. Furthermore, it suggests that NK cells can lose inhibition in the presence of normal MHC class I, simply by changing the peptide content of cognate MHC class I molecules. Indeed, small changes in peptide content of MHC class I can differentially affect KIR2DL2 and KIR2DL3-positive NK cells when antagonist peptides are present (15). This may be important because KIR2DL3, but not KIR2DL2, is associated with protection against hepatitis C virus infection (16).

To date, peptide antagonism has been defined only for HLA-C^*^0102, endogenously expressed in TAP-deficient cells, using a peptide variant of VAPWNSLSL (VAPWNSDAL and VAPWNSDYL) (12, 13). To determine whether this phenomenon can be broadened to other peptide:HLA combinations, we have now studied HLA-C^*^0304. Here we show that peptide antagonism is not restricted to one HLA-C allele, but is likely to be a more generalizable phenomenon for KIR2DL2/3. Since the observation of peptide antagonism in inhibitory KIRs, two different mechanisms have been proposed to explain the phenomenon (17). In one model, specific biochemical modifications induced by antagonist peptides in very upstream signaling events, such as the dephosphorylation of tyrosine residues in ITIMs by phosphatases recruited by antagonist ligand-receptor complexes has been implicated. This is similar to antagonism in TCR signaling (18–20). The other model suggests disruption of tight spatial clustering of inhibitory receptors in the presence of antagonist peptides should play a dominant role in inducing antagonistic signals. We developed a spatially resolved *in-silico* model to investigate the proposed mechanisms that underlie peptide antagonism in KIRs. In particular, we find that changing the nature (tight to diffuse) of KIR clusters plays a driving role in mediating suppression of inhibitory signals by the antagonist peptides.

## Materials and Methods

### Peptides

Synthetic peptides were purchased from Peptide Protein Research (Hampshire, UK) and GL Biochem (Shanghai, China). Their identities were confirmed by HPLC and MS and purity was >95%.

### Cells Lines, PBMC and Cell Culture

721.221C^*^03-ICP47 cells are MHC class I negative 721.221 cells transduced with HLA-C^*^0304 and ICP47 to block TAP and permit exogenous peptide loading. 721.221C^*^03-ICP47 cells were cultured in R10 medium (RPMI 1640 medium supplemented with 1% penicillin/streptomycin [Invitrogen] and 10% FBS [HyClone]) and 500 μg/ml of Hygromycin (HygroGold, Invivogen, Toulouse, France). NKL lines were transfected with the KIR2DL3-GFP receptor construct (NKL:2DL3-GFP) (13) and cultured in R10 medium supplemented with 100U/ml of IL-2. NKL:2DL3-GFP and target cells were washed and re-suspended in AIM-V+AlbuMAX (AIM-V) (BSA) 1X medium (Gibco Life Technologies, Paisley, UK) before co-culture. All cells were maintained in culture at 37°C, 5%CO_2_ and in humidified atmosphere.

### Peptide Stabilization Assay

2x10^5^ 721.221C^*^0304-ICP47 cells were incubated overnight at 26°C, 5%CO_2_ in R10 alone or in R10 medium containing 0–100 μM of the specified peptide. Stabilization was assessed with the W6.32 antibody which recognizes HLA-A, -B, and -C and the DT9 antibody which recognizes HLA-C. Both primary antibodies were produced in-house. After incubation with the peptides, cells were washed twice with wash buffer (PBS 1X + 1%BSA + 0.1%NaN_3_) and re-suspended in blocking buffer (wash buffer + 10%human AB serum) then incubated for 30 min at 4°C. Cells were then incubated at 4°C with these antibodies for 1 h followed by 30 min of incubation with a polyclonal goat anti-mouse antibody conjugated with PE diluted at 1/50 (Abcam, UK), washed, then re-suspended in fixing buffer [1x PBS+ 1%PFA (Santa Cruz, USA)] and analyzed on a BD Accuri C6 Flow Cytometer with BD CFlow Software (BD Biosciences, Oxford, UK). Ten thousand live events were collected.

### Measurement of the Decay of Cell Surface HLA-C Molecules

6 × 10^5^ 721.221-ICP47 cells were pulsed with 100 μM of peptide and incubated overnight at 26°C. 1 × 10^5^ cells per condition were then harvested resuspended in 100 μl R10 containing 5 μg/ml brefeldin A (Biolegend, San Diego, USA) at 37°C for various time points. Surface expression of HLA-C was quantified by staining using DT9 followed by PE-labeled goat anti mouse IgG (Abcam, UK) and analyzed by flow cytometry.

### Degranulation Assays

Human PBMC were isolated from the blood of 8 healthy donors using Hypaque-Ficoll (GE Healthcare, Amersham, UK) density centrifugation, with informed consent and full ethical approval (NRES reference: 06/Q1701/120). 3 × 10^5^ PBMCs were stimulated overnight with 1 ng/mL recombinant human IL-15 (R&D Systems). Peptide pulsed 721.221C^*^0304-ICP47 targets were prepared as for the stabilization assays. Target cells were resuspended with PBMCs at an effector-to-target (E:T) ratio of 5:1 in fresh R10 medium containing peptide and anti-CD107a-efluor-660 antibody (eBioscience, Hatfield, UK). Cells were incubated for 1 h at 26°C, then 6 μg/mL Golgi- Stop™ (BD Biosciences) was added, and incubated for a further 4 h at 26°C. Cells were washed, blocked with blocking buffer for 30 min and then stained with the following antibodies: anti-CD3-PerCP (Biolegend, San Diego, USA), anti-human CD56-PE, and anti-human KIR2DL2/L3/S2, CD158b-FITC (both BD Biosciences). Cells were fixed in 1% PFA and analyzed by flow cytometry. Individual assays for each donor were performed once in duplicate and the mean value used for subsequent analysis.

### Peptide Elution From MHC Class I and HPLC Analysis

Peptide elution from class I molecules was performed by mild acid elution (21). 2.5 × 10^6^ 721.221C^*^0304-ICP47 cells were incubated overnight with 20 μM of peptide, cells were harvested and washed three times. Cell pellets were re-suspended in 250 μl citrate-phosphate buffer (0.131M Citric Acid + 0.066M Sodium Phosphate Dibasic, pH 3.3), gently mixed and incubated for 1 min at room temperature, before being centrifuged at 16,000g to eliminate any trace of cells debris in the supernatants. The clear supernatant was retained, diluted with loading buffer (98% H_2_O + 2% ACN + 0.05% TFA) and analyzed by uHPLC. Eluted peptides were separated by a one dimension set up Reversed Phase-HPLC (RP-HPLC) on a Dionex system using a reversed phase C18 trapping and analytical columns using an acetonitrile gradient in 0.05% TFA. Loading buffer, solvent A (100%H_2_O + 0.05% TFA) and solvent B (20%H_2_O + 80% ACN + 0.05% TFA) were used for the UHPLC analysis and peptide detection. H_2_O, Acetonitrile (ACN) and Trifluoroacetic acid (TFA) were all HPLC grade and from Fisher Scientific (Loughborough, UK). Peptides were detected by a UV detector at a wavelength of 214 nm.

### KIR-Fc Binding Assay

2 × 10^5^ 721.221:C^*^0304-ICP47 cells were incubated in the presence or absence of 100 μM peptide overnight at 26°C. rhKIR2DL2-Fc chimera (R&D systems, Abingdon, UK) was conjugated with protein A Alexa Fluor 488 (Invitrogen, Paisley, UK) at a ratio of 12:1 KIR: protein A overnight at 4°C. The following day peptide loaded cells were stained with 50 μg/ml of KIR-Fc for 1 h at room temperature. Samples were washed twice with PBS and bound fusion proteins detected by flow cytometry.

### Confocal Microscopy

721.221:C^*^0403-ICP47 cells were cultured with 100 μM final concentration of peptide overnight at 26°C, then incubated at a 1:1 ratio with NKL:2DL3-GFP cells for 10 min at 37°C. Conjugates were fixed in 2% PFA and imaged by resonance scanning confocal microscopy (TCS SP8, Leica). Images were acquired with Leica Application Suite X (Leica) and analyzed with ImageJ (National Institutes of Health) software. The increase in fluorescence intensity at the immune synapse was calculated as a ratio of the average fluorescence intensity along the interface of NKL:2DL3-GFP cells with 721.221:C^*^0403-ICP47 cells, compared with the total membrane GFP signal, with both values corrected for background fluorescence, as measured within an empty region of the image.

### Mathematical Modeling

We developed two (referred to as Models 1 and 2) spatially resolved *in-silico* models of membrane proximal NK cell signaling in order to investigate possible mechanism behind peptide antagonism. The simulation box (*L*x*L*x*l* = 2 × 2 × 0.02 μm), representing the interface between the plasma membrane and the cytosol, was divided into small chambers (shown by gray grids in Figure S2) of size *l*^3^ such that the signaling molecules were well-mixed inside the small chambers. The signaling molecules were modeled as agents that diffused on this 3-dimensional grid. The diffusion of the molecules between neighboring chambers was treated as reactions occurring at rates D/*l*^2^. Membrane bound and cytosolic molecules diffused with rates 0.01 μ m^2^/s and 10.0 μm^2^/s, respectively. Upon encounter, they reacted (Table S1) with one another following the law of mass action. The spatially resolved simulations containing biochemical signaling reactions with intrinsic noise fluctuations and diffusive processes were performed using a software package called Stochastic Simulation Compiler (SSC) (22) that uses Gillespie algorithm (23).

For both the models, the inhibitory KIR2DL2/3 receptors were localized in a preformed micro-cluster of size 0.2 × 0.2 μm^2^ at the center of the simulation box. This size is slightly larger but is in the same order of magnitude in diameter (~0.08 μm) as KIR2DL1 nanoclusters observed by Oszmiana et al. (24). Dissociation constants K_D_ (= k_off_/k_on_) for the binding of KIR2DL2/3 with the inhibitory ligands (HLA-C^*^0304:L7R) and the antagonist peptide (HLA-C^*^0304:L7D) were estimated to be K_D_ = 0.8 μM and K_D_ = 4.4 μM, respectively, using staining of KIR2DL2-Fc by the inhibitory peptide L7R and the antagonist peptide L7D. Our simulations probed two different cases: 1: KIR2DL2/3 was stimulated by strong inhibitory ligands (L7R) whose concentration was gradually increased from 0 to 12.5 micromolar and 2: the same set of receptors stimulated by both L7R and weak affinity antagonist peptides (L7D) such that the total concentration of L7R+L7D was kept constant at 12.5 μM. In the presence of L7D we increased the cluster size of KIR2DL2/3 such that the total numbers of KIR2DL2/3 and SFKs in the cluster remained the same for all the cluster sizes (Figure S3) (further details in the Supplemental Material). The system reached steady state within 5 min for all ligand concentrations used in this study. We have calculated the steady state pVav1 abundances by calculating the time average of pVav1 abundance between 5 and 15 min. The parameter values used are shown in Tables S1–S3. The SSC codes and the input files are available at https://doi.org/10.5061/dryad.mg3n2c2.

## Results

### Allelic Diversity in HLA-C Binding Peptides

**Figure 1:**
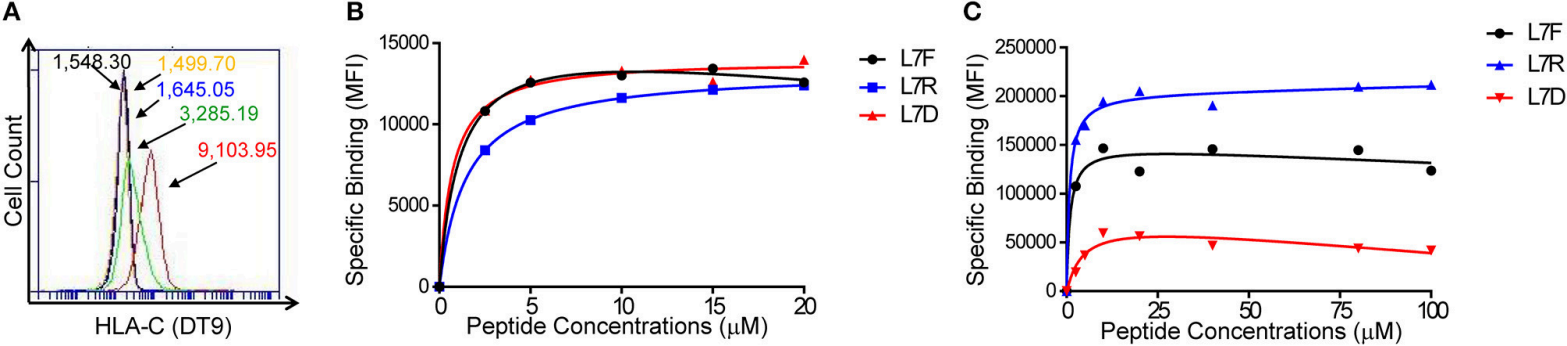
Peptide stabilization and KIR2DL2 binding on 721.221:C*0304-ICP47 cells after peptide loading. **(A)** HLA-C expression measured by flow cytometry using DT9 antibody. Histogram plots show unstained (black), staining with secondary antibody only (yellow) 721.221 (blue), 721.221C*0304 (red) and 721.221:C*0304-ICP47 (green). Numbers correspond to the MFI. **(B)** Stabilization of HLA-C*0304 after peptide loading with GAVDPLFAL (L7F), GAVDPLRAL (L7R), GAVDPLDAL (L7D) and analysis by flow cytometry using DT9 antibody. MFI values were corrected by subtracting the MFI value obtained in absence of peptide. **(C)** KIR2DL2 binding after loading 721.221:C*0304-ICP47 cells with different peptides. Cells loaded with GAVDPLFAL (L7F), GAVDPLRAL (L7R), GAVDPLDAL (L7D) were stained with KIR2DL2-Fc to assess the KIR binding. MFI values were determined by subtracting the MFI value obtained in absence of peptide from the observed value for each condition.

We transfected 721.221:C^*^0304 cells with ICP47 (221:C^*^0304:ICP47 cells) to block TAP and enable exogenous peptide loading. Successful transfection was measured by a decrease in HLA-C expression at the cell surface indicating that the ICP47 protein was functional (Figure 1A). Our previous work has shown that peptide antagonism is determined by the residue at position (p7) of the HLA-C bound peptide (12). Using the HLA-C^*^0304 binding peptide GAVDPLLAL, we tested a series of peptides with the following modifications at p7: GAVDPLRAL (L7R), GAVDPLFAL (L7F), and GAVDPLDAL (L7D) (7, 25). Based on our previous work using an HLA-C^*^0102 binding series of peptides we anticipated that L7F would be a strong inhibitor, L7R a weak inhibitor and L7D antagonistic. We loaded the peptides onto 221:C^*^03:ICP47 cells and measured HLA-C stabilization (Figure 1B). All variants of GAVDPLLAL peptides with a modification at p7 stabilized HLA-C on the cell surface to similar extents. Binding assays using a KIR2DL2-Fc construct showed that this series of peptides have different affinities for KIR2DL2 (Figure 1C). Peptide binding for the KIR2DL2 construct were higher for L7R and lower for L7F and L7D.

**Figure 2:**
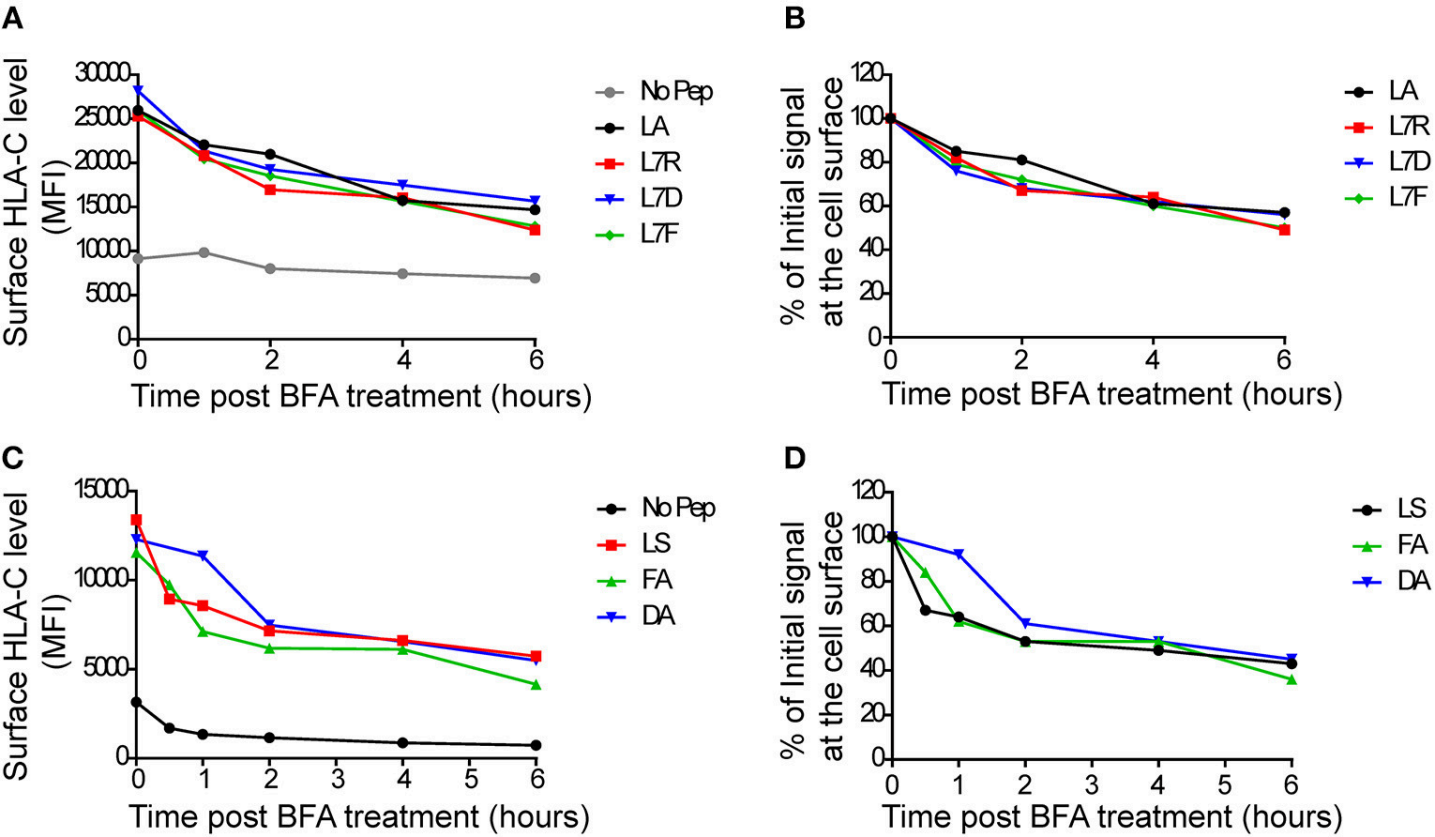
Decay of cell surface HLA-C following peptide loading. **(A)** 721.221:C*0304-ICP47 cells were loaded overnight with 100 μM of GAVPDLLAL p7 derivative peptides then treated with brefeldin A. Aliquots harvested after 0, 1, 2, 4, and 6 h were then stained with DT9 antibody (p7 residue modifications are indicated in the key) **(B)** This panel shows the MFI data from **(A)** normalized to the initial HLA-C surface level (MFI at t = 0 h). **(C)** 721.174 cells were loaded overnight with 100 μM of HLA-C*0102-binding VAPWNSXXL derivative peptides then treated with brefeldin A. Aliquots harvested after 0, 1, 2, 4, and 6 h were then stained with DT9 antibody (p7 and p8 residues are indicated in the key). **(D)** This panel shows the MFI data from **(C)** normalized to the initial HLA-C surface level (MFI at t = 0 h). Similar rates of decay were observed in two separate experiments.

To assess MHC class I stability in the context of the different peptides, we performed a series of brefeldin-A peptide decay assays. We observed that the HLA-C^*^0304 peptides with p7 variants had similar decay kinetics to the other peptides (Figures 2A,B). However, the previously studied HLA-C^*^0102 antagonist peptide VAPWNSDAL (VAP-DA) (12) had a slower decay compared to that of the strong inhibitory peptide VAPWNSFAL (VAP-FA) (Figures 2C,D). In our previous publications (12, 13) the VAP-DA peptide was described as being an antagonistic peptide that disrupted NK cell inhibition induced by VAPWNSFAL. This suggested that this change in inhibition is because VAPWNSDAL is more stable and hence prevents loading of VAPWNSFAL onto HLA-C^*^0102. However, this was not the case for HLA-C^*^0304, for which stability was equivalent for all peptides tested.

**Figure 3:**
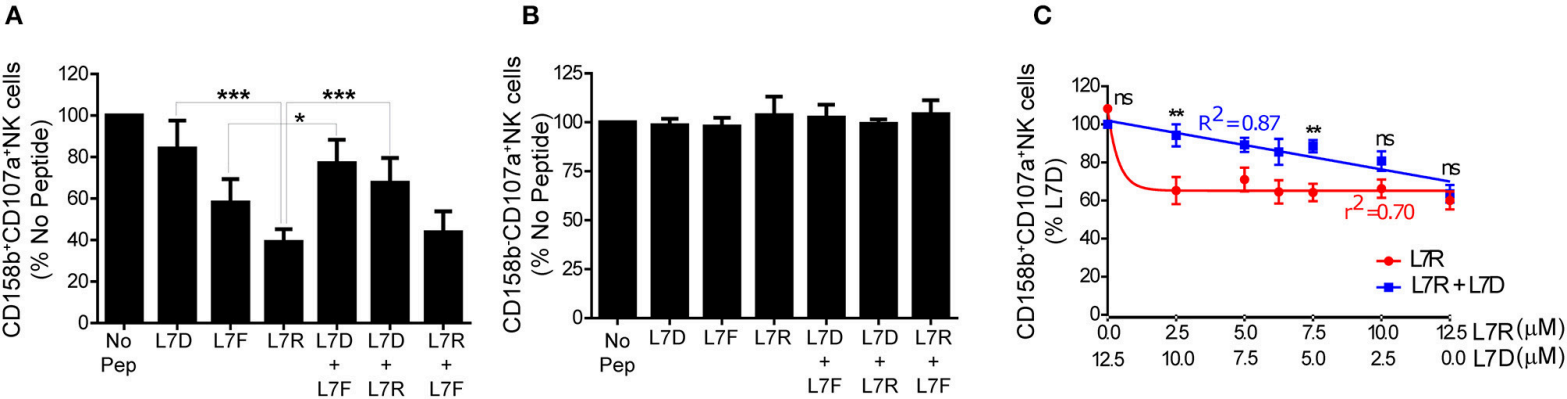
Inhibition of KIR2DL3+ NK cells by p7 derivatives of GAVPDLLAL, alone or in combination. **(A,B)** Degranulation of KIR2DL3^+^ **(A)** and KIR2DL3^−^**(B)** NK cells in response to the p7 variants of GAVPDLLAL, GAVPDLA**D**AL (L7D), GAVPDL**F**AL (L7F) and GAVPDL**R**AL (L7R). 721.221:C*0304-ICP47 cells were incubated overnight with the indicated peptides at 12.5 μM and then used as targets in degranulation assays with IL-15-activated NK cells. Degranulation of CD158b^+^ (KIR2DL3^+^) NK cells was measured. Data are expressed as mean percentages +/-SEM from 8 independent experiments. All values have been normalized to degranulation in the absence of peptide (*p < 0.05 and ***p < 0.001 using two way ANOVA for the indicated comparisons). **(C)** Degranulation of KIR2DL3^+^ NK cells in response to 721.221C*0304-ICP47 cells incubated with different combinations of L7D and L7R peptides. Data are expressed as mean percentage +/-SEM and normalized to the value observed with 100% L7D (**p < 0.01 using two- way ANOVA). Results are representative of 4 independent experiments using 6 different donors.

We then tested inhibition of KIR2DL3-positive NK cells using a CD107a degranulation assay. We studied eight different KIR2DL3 homozygous donors and found that there was consistently greater inhibition of KIR2DL3+ NK cells by the peptide with arginine (L7R) compared to phenylalanine (L7F) at p7 (*p* < 0.01; Figure 3A). L7D behaved as a peptide antagonist for both L7R and L7F, similar to the p7 aspartate modified peptide for HLA-C^*^0102 (Figure 3A and Figure S1). Additionally, no differences were observed for KIR-negative NK cells (Figure 3B and Figure S1). These results are consistent with the KIR binding results using a KIR2DL2-Fc fusion construct, in that L7R had stronger binding for KIR2DL2 than L7F. However, this contrasts with HLA-C^*^0102 and VAPWNSXAL for which arginine at p7 was a weaker inhibitor of KIR2DL3-positive NK cells as compared to phenylalanine (12).

Peptide antagonism by HLA-C^*^0102 using agonist and antagonist peptide combinations gives a linear relationship between inhibitory peptide concentration and NK cell inhibition, as opposed to a one-phase decay curve obtained using the inhibitory peptide alone. We consequently performed peptide titration experiments where the concentration of L7R varied from 12.5 to 0 μM whilst the L7D concentration was increased from 0 to 12.5 μM (Figure 3C). Our results showed that for all concentrations of L7D tested, there was a release of NK cells from L7R–mediated inhibition. Moreover, we observed that a low concentration of L7R (2.5 μM) was enough to induce almost 50% NK cell inhibition in the absence of L7D as opposed to 80% in the presence of L7D. Furthermore, the change in inhibition due to the combination of L7R and L7D was linear, consistent with our previous peptide antagonism data for HLA-C^*^0102 (12). Thus, at 0.625 μM L7R there was 40% inhibition of KIR2DL3+ NK cells in the absence of L7D, but in the presence of L7D this level of inhibition was present at a more than 10-fold greater concentration of L7R (7.5 μM). Thus, L7D is a weak KIR binding peptide that alone does not induce inhibition of KIR2DL3-positive NK cells but disrupts the inhibition driven by L7R.

### L7D Induced Reversal of Inhibition Is Not Due to Peptide Displacement

**Figure 4:**
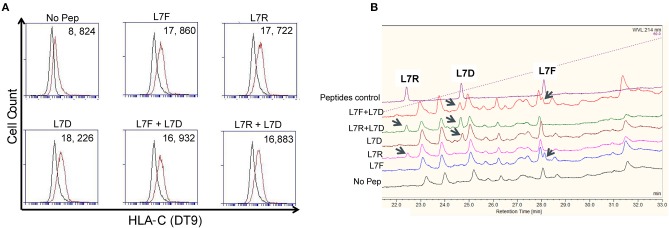
The antagonist peptide L7D does not displace inhibitory peptides L7F and L7R. 721.221C*0304-ICP47 cells were loaded with single peptides or a combination of L7F + L7D or L7R + L7D. Cells were washed three times prior to peptide elution and subsequent HPLC analysis. **(A)** shows stabilization of HLA-C*0304 as determined by DT9 staining following washing but before mild acid elution. The “No peptide” histogram compares stained with unstained cells; all other histograms compare stabilization in the presence of peptide with the “No peptide” control. MFI values are indicated in the top right. Also upper panel has a number on top left of each plot. **(B)** Analysis of eluates from 721.221C*0304-ICP47 cells by HPLC. Eluted peptides were analyzed via HPLC on a 4–60% acetonitrile gradient. Results are representative of 2 experiments.

To exclude the possibility that the “antagonism” effect observed was due to preferential presentation of the L7D peptide, we analyzed the binding of peptides to MHC class I by HPLC. Cells were pulsed overnight with 20 μM peptide and washed until no peptide was detectable in the wash by HPLC. DT9 staining confirmed effective stabilization of HLA-C expression on the cell surface following these washes (Figure 4A). HPLC results showed that all the peptides loaded could subsequently be eluted from MHC class I (Figure 4B). When we mixed L7R+L7D or L7F+L7D, both peptides were detectable after acid elution. Furthermore, the peak height showed that peptides in combination were similarly loaded onto HLA-C and thus L7D does not significantly displace L7R or L7D from the cell surface. Thus, the effect driven by L7D peptide was not due to peptide displacement.

### L7D Disrupts KIR Aggregation at the Immunological Synapse

**Figure 5:**
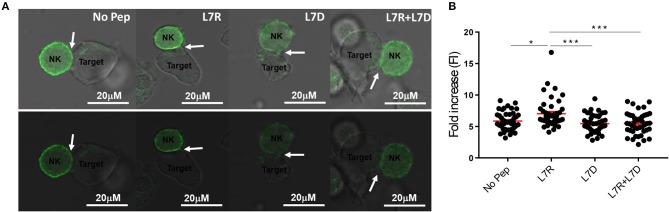
KIR2DL3 aggregation at the surface between target and effector cells induced by the different peptides. **(A)** Confocal microscopy images of target and NKL:2DL3 cell conjugates. 721.221C*0304-ICP47 cells were loaded with the indicated GAVPDLLAL p7 derivative peptides at a concentration of 12.5 μM, incubated with NKL:2DL3-GFP cells and then analyzed by confocal microscopy. Comparison of GFP intensity at the interface between 721.221C*0304-ICP47 target cells loaded with 12.5 mM of indicated peptides and NKL:2DL3 cells. Arrows indicate the interface between target and effector cells. Scale bar: 20 μM. **(B)** The fold increase in GFP fluorescence intensity at the interface between target and effector cells was compared with total GFP of the NKL:2DL3-GFP cell membrane and then plotted for the different conditions. 100–160 conjugates were analyzed and L7R was compared to other conditions using a one way ANOVA. *p < 0.05, ***p < 0.001. The means ± SEM are shown in red.

We next compared inhibitory synapse formation in the presence of L7D, L7R, or L7R+L7D. Analysis of KIR receptor clustering at the immune synapse using a GFP-linked KIR2DL3 protein, showed that L7R, but not L7D induced increased aggregation of KIR2DL3 (Figures 5A,B). However, in the presence of the L7D peptide no increase in aggregation of KIR2DL3 by L7R was observed, consistent with the disruption of inhibition noted in the degranulation assays.

### *In silico* Modeling Reveals Co-clustering of Src Family Kinases and KIRs Is key to Induce KIR Antagonism

**Figure 6:**
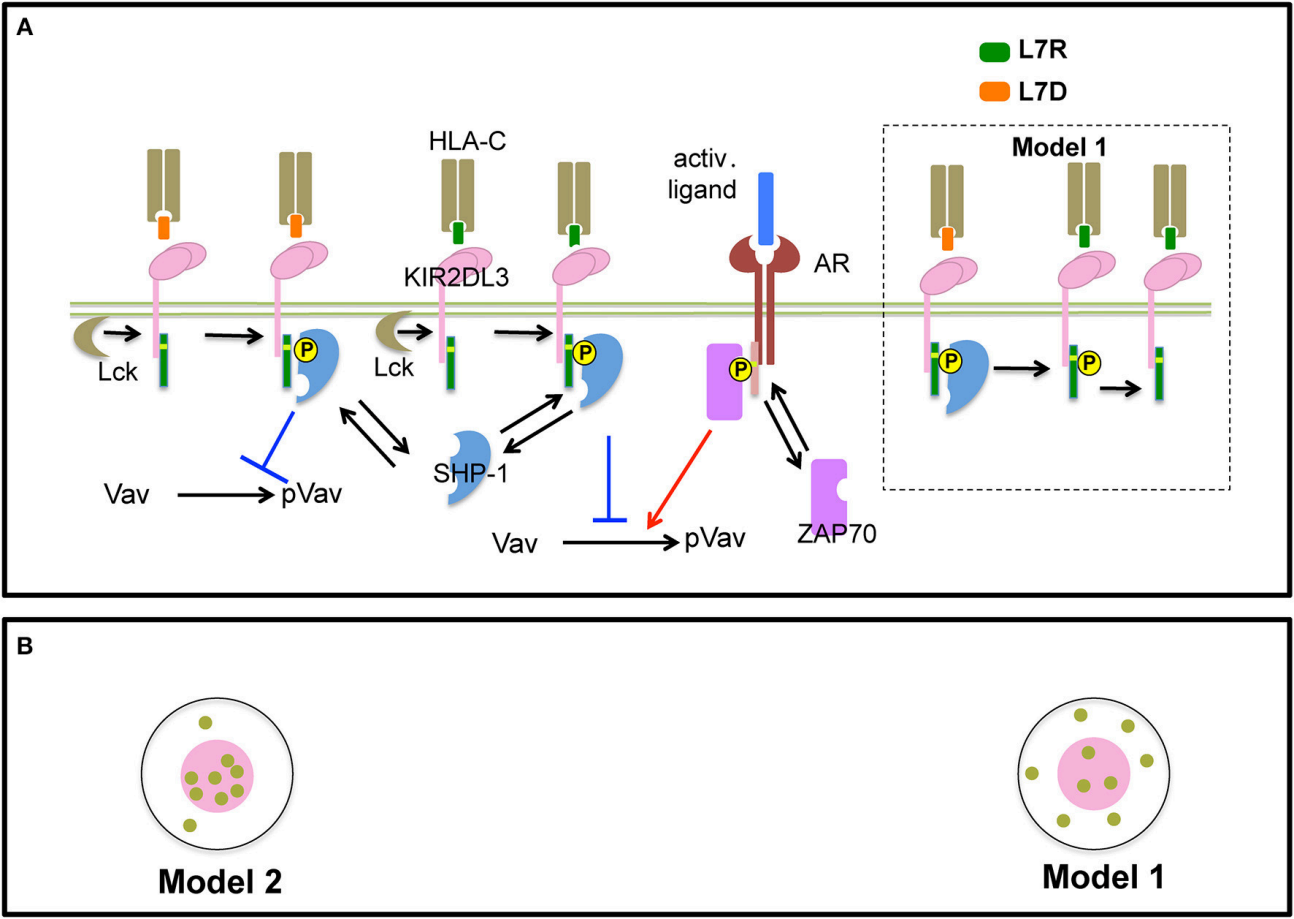
Schematic representation of processes in Model 1 and 2: **(A)** Inhibitory KIR receptors bind to HLA-C molecules bound to inhibitory (L7R, green) or antagonist (L7D, orange) peptides. The binding affinity of KIR to HLA-C:L7D is four times lower than to HLA-C:L7R. After ligation, SFKs (e.g., Lck) phosphorylate the cytoplasmic tail of KIRs which bear an ITIM. We simplify our model by assuming that an ITIM can reside in only two states of activation: unphosphorylated or fully phosphorylated (marked by a yellow circle). Phosphorylated ITIMs recruit cytosolic phosphatase SHP-1. ITIM bound SHP-1 then binds and de-phosphorylates p-Vav. The activating receptor (AR) binds to unspecified activating ligands. SFKs phosphorylate ITAMs associated with activating receptors bound to their cognate ligands. Similar to ITIMs, ITAMs exist in two states, unphosphorylated and phosphorylated (marked by a yellow circle). Phosphorylated ITAMs dock cytosolic Syk family kinase called Zap-70. ITAM bound Zap-70 phosphorylates and activates Vav-1. The Vav activation/de-activation module is the same for both Model 1 and Model 2. In addition, in Model 1, antagonist-HLA-KIR ITIM bound SHP-1 molecules dephosphorylate neighboring phospho-ITIMs associated with KIRs bound to both inhibitory and antagonist ligands (shown inside the dotted black box). **(B)** SFKs do not co-cluster with KIRs in the KIR2DL3 microclusters in Model 1, whereas, SFKs and KIRs co-cluster in Model 2.

In order to gain insights into a potential mechanism for peptide antagonism, we developed two spatially resolved computational models, Model 1 and Model 2. The two models were based on hypotheses proposed by Rajagopalan and Long to explain KIR antagonism (17) and describe membrane proximal signaling events that occur at early timepoints involving HLA-peptide ligands, inhibitory KIR (KIR2DL3/KIR2DL2) molecules, Src family kinases (SFKs), Syk family kinase (Zap-70), phosphatase SHP-1, and the guanine nucleotide exchange factor (Figures 6A,B). The two models contain common signaling reactions and spatial clustering of inhibitory KIRs and differ in terms of a specific reaction involving antagonist peptides and co-clustering of inhibitory KIRs and SFKs. We provide details regarding the similarities and differences between Model 1 and Model 2 below and a summary of these details are shown in Table 1.

**Table 1:** Summary of the similarities and differences between Model 1 and 2.

	**Clustering of inhibitory KIRs alone**	**Inhibitory KIR + SFK co-clustering**	**pITIM de-phosphorylation by SHP-1 bound to antagonist complex**	**Canonical biochemical signal integration via Vav1**
**Model 1**	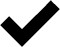	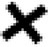	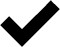	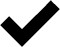
**Model 2**	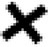	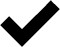	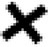	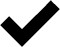

First, we describe biochemical reactions and spatial clustering of inhibitory KIRs that are common to both the models. In these models, plasma membrane bound inhibitory KIRs are associated with immunotyrosine based inhibitory motifs (ITIMs). The tyrosine residue pairs in ITIMs are phosphorylated by SFKs upon inhibitory KIRs binding to HLA-inhibitory (L7R) or –antagonist (L7D) peptide ligands. The SFKs reside in the plasma membrane and the unbound SFK molecules diffuse freely in the plasma membrane. Phosphorylated ITIMs recruit SHP-1 to the inhibitory KIR-ligand complex and the bound SHP-1 dephosphorylates phosphorylated Vav1 (pVav1) following an enzymatic reaction (26). The unbound SHP-1 and Vav1 molecules reside and diffuse freely in the cytosol. An activating NK receptor, which is not characterized specifically in the model, is associated with immunotyrosine based activation motifs (ITAMs). The activating NK receptor binds to an unspecified activating ligand and upon formation of the ligand-receptor complex, the tyrosine residues in the ITAMs are phosphorylated by the SFKs. The phosphorylated ITAMs act as docking sites and recruit cytosolic Zap-70 to the activating NK receptor complex. Zap-70 in turn phosphorylates Vav1. Thus, Vav1 phosphorylation is a product of the integration of activating and inhibitory signals and the abundance of pVav1 is used as a marker for NK cell activation (26, 27). For both of our models, activation states of ITIMs or ITAMs have been approximated to two states: *active* and *inactive*. The *active* state represents both partially and fully phosphorylated ITIM/ITAM states and the *inactive* state denotes ITIMs/ITAMs devoid of any tyrosine phosphorylation. The diffusion rate in plasma membrane (D ~10^−2^ μm^2^s^−1^) is 1000 times smaller than that of the cytosol (D ~10 μm^2^s^−1^) (28). Inhibitory KIRs are clustered in a microcluster in both the models (Figure 6B and Figure S2). Previous experiments showed that in the presence of antagonist peptides the tight clustering of KIRs is disrupted (13). This behavior was implemented in the simulation by increasing the inhibitory KIR cluster size but decreasing the density of KIRs within the cluster as the concentration of antagonist peptides L7D was increased (see Figure S3). Therefore, in our simulations as the concentration of L7D increased, the same number of KIR molecules were distributed evenly in larger cluster sizes. The KIR cluster size was assumed to be independent of inhibitory peptide (L7R) concentration.

The differences between Models 1 and 2 are as follows. In Model 1, SHP-1 bound to the antagonist-peptide HLA:inhibitory KIR complex dephosphorylates pITIMs of neighboring KIR-peptide-MHC complex. Therefore, in Model 1, antagonists disrupt inhibitory signals by this reaction. This reaction is absent in Model 2. The next difference arises in terms of co-clustering of inhibitory KIRs and the SFKs. In Model 1, SFKs are not co-clustered with the inhibitory KIRs in the microclusters, whereas, in Model 2, SFKs and inhibitory KIRs are co-clustered in the microclusters (Figure 6 lower left and Figure S2). The experimental justification behind co-clustering of inhibitory KIRs and the SFKs is provided by the confocal imaging experiments of Treanor et al. (24, 29). Treanor et al. demonstrated that in YTS/KIR2DL1 cells interacting with 221/Cw6 target cells, the YFP tagged Lck co-localizes with inhibitory KIR2DL1 receptors at the immunological synapse.

The differences between Models 1 and 2 in biochemical reactions and spatial clustering of the inhibitory KIRs and SFK probed the relative roles of these contributions in suppressing of inhibitory signals by antagonistic peptides. In Model 1, the primary mode of suppression of the inhibitory signals by antagonist peptides is via the dephosphorylation of pITIMs by the SHP-1 bound to the antagonist peptide:inhibitory KIR complex. In contrast, in Model 2, the antagonist peptides repress the inhibitory signals by disrupting the tight co-clustering of inhibitory KIRs and the SFKs which lowers the propensity for the SFKs to phosphorylate ITIMs. Since the inhibitory KIRs and the SFKs are not co-clustered in Model 1, the effect of the disruption of the KIR microclusters due to the antagonist peptides relating to the ability of the SFKs to phosphorylate the ITIMs is weak. Therefore, Model 1 proposes a biochemical origin whereas Model 2 proposes an alteration of spatial clustering based mechanism for the observed KIR antagonism (Table 1).

**Figure 7:**
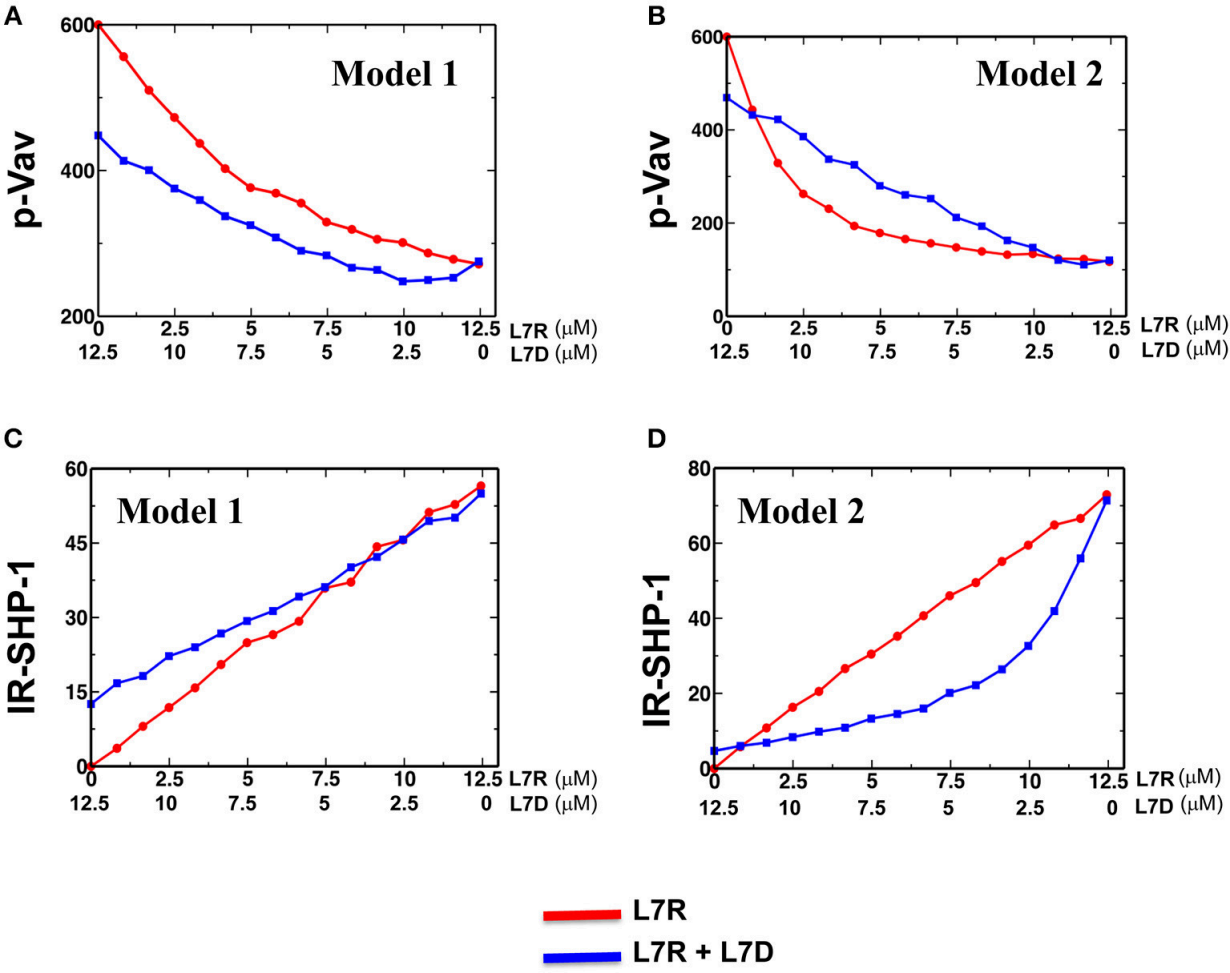
Activation and inhibition in Model 1 and 2. **(A)** and **(B)** Variation of abundance of pVav in steady state in Model 1 **(A)** and Model 2 **(B)** as the concentration of the single (red) inhibitory peptide (L7R) or the mixture (blue) of inhibitory (L7R) and antagonist (L7D) peptides is increased. The L7R concentration in the single or mixed peptide stimulation changes from 0 to 12.5 μM. The L7D concentration is adjusted such that the mixture concentration is always at 12.5 μM. The x-axis shows the L7R concentrations for the single peptide simulation and L7D concentration for mixed peptide simulation. In this scheme, a value of x = 5 μM L7R indicates a single peptide stimulation with 5 μM L7R and a 7.5 μM L7D right underneath it indicates a mixed peptide stimulation with 5 μM L7R + 7.5 μM L7D. **(C)** and **(D)** Variation of abundance of SHP-1 bound to the ITIMs for Model 1 **(C)** and Model 2 **(D)** with the concentration of ligands (single or mixed peptide) for the cases shown in **(A,B)**. For all the figures a K_R_ value of 5 μM was used.

We carried out simulations for both the models in two scenarios where the inhibitory KIRs were presented (i) with inhibitory peptides bound to HLA or (ii) with a mixture of inhibitory and antagonist peptides bound to HLA. The concentration of the activating ligands were fixed to the same value for both the models. Similar to the experiments, the number of the inhibitory peptide-HLA molecules was held fixed in the single and mixed peptide stimulations, but the proportion of the antagonist peptide-HLA was increased. In both the models, as the proportion of the antagonist peptide-HLA ligands increased inhibitory KIR clustering was made more diffuse and consequently the inhibitory KIR density inside the microcluster decreased with increasing antagonist concentrations. However, the SFKs were not co-clustered with the inhibitory KIRs in Model 1 and the spatial distribution of the SFK molecules were not altered in the model as the KIR density was decreased with the increasing dose of the antagonist peptide. In contrast, the SFKs were co-clustered with the KIRs in Model 2, and the densities of both SFKs and KIRs in the microclusters decreased in the model as the antagonist dose was increased. The disruption in KIR microcluster with an increase in antagonist peptide concentration and consequent decrease in KIR density within the microcluster was implemented by hand, without taking into account any detailed molecular mechanism behind it. pVav1 abundances reached a steady state at ~5 min in the simulations and the steady state pVav1 values were compared (Figures 7A,B) between the models. pVav1 abundances decreased monotonically in both Model 1 (Figure 7A) and Model 2 (Figure 7B) as the number of the inhibitory peptides increased in the single peptide stimulations. However, when the numbers of inhibitory peptides were increased by the same amounts in the mixture containing inhibitory and antagonist peptides, and the density of the KIR molecules in the cluster was decreased as shown in Figure S3, then the pVav1 abundance decreased more than that of the single peptide stimulation for Model 1 (Figure 7A) displaying increased inhibition instead of an antagonistic behavior. In contrast, increasing the inhibitory peptide numbers in the mixed peptide simulation in Model 2 produced a slower decrease of pVav abundances where the pVav abundances in the mixed peptide stimulations were larger than that of the single peptide stimulation for a range of inhibitory peptide concentrations (Figure 7B). The density of the KIR molecules was also decreased in Model 2 as shown in Figure S3. Thus, Model 2 displayed peptide antagonism which is qualitatively similar to that observed in experiments for the L7R peptides (Figure 3C).

The above behavior can be explained in the following way. In Model 1, the presence of antagonist peptides made the KIR clustering diffuse, and consequently, despite the recruitment of more SHP-1 by the ITIMs in the presence of antagonists (Figure 7C), the pITIM bound SHP-1 has access to few neighboring pITIM to act upon. Therefore, the antagonist peptide bound KIRs were not efficient in relieving inhibition by the inhibitory peptides. In Model 2, because the inhibitory KIRs and the SFKs were co-clustered, the diffuse clustering of KIR in the presence of the antagonist peptides increased the search time for the SFKs to find and phosphorylate ITIM molecules in the neighborhood, and consequently less SHP-1 was recruited to the KIR complexes (Figure 7D) compared to that in single inhibitory peptide stimulations which produced tight co-clustering of KIRs and SFKs. Therefore, Model 2 reproduced the peptide antagonism observed in the experiments and demonstrated that the suppression of KIR inhibition by the antagonist peptides was a direct consequence of change in KIR clustering due to the presence of antagonist peptides.

The extent of increase in the inhibitory KIR cluster size as the concentration of the antagonist peptide increased is an important factor in enabling Model 2 but not Model 1 to generate the KIR antagonism. For example, if the KIR cluster size remains fixed or changes by a small amount (3 times across the range of peptide concentration used) as the antagonist concentration changes from 0 to 12.5 μM, Model 1 is able to generate antagonistic behavior (Figure S4). Since a change in cluster size is the only driver of antagonism to inhibition in Model 2, implementing small changes in the KIR cluster size jeopardizes Model 2's ability to generate peptide antagonism. However, Borhis et al. (13) showed a large increase in the inhibitory KIR2DL3 cluster size as the antagonist peptides were mixed with inhibitory peptides. Thus, Model 2 appears to be more consistent with the observed peptide antagonism compared to Model 1.

## Discussion

We previously identified the phenomenon of peptide antagonism for HLA-C^*^0102 using peptide variants of VAPWNSLSL. We have investigated this mechanism further by studying HLA-C^*^0304 and derivatives of the peptide GAVPDLLAL. Aspartate at p7 for both peptides generated antagonist peptides, although for VAPWNSDAL this generated a more stable peptide MHC complex than the inhibitory peptide with phenylalanine at position 7. Conversely GAVPDLDAL had a similar off rate to both GAVPDLRAL and GAVPDLFAL, and did not displace either of these peptides as determined by the peptide stabilization assays. Consistent with GAVPDLDAL being an antagonist, at a 50:50 ratio of inhibitory (GAVPDLRAL) to antagonist peptide (GAVPDLDAL) the level of tight clustering at the immune synapse was reduced to that of the antagonist peptide alone, which is relevant as tight clustering of KIR molecules is associated with a productive inhibitory signal (30, 31).

We investigated the roles of KIR spatial clustering and inhibitory signals initiated by interaction of HLA-C bound antagonistic peptides and KIRs using mathematical modeling. We developed two models, Model 1 and Model 2, to evaluate the role of spatial clustering of KIR and SFKs in giving rise to the peptide antagonism. Our simulations found that Model 2 is able to describe the suppression of KIR inhibition by the antagonist peptide L7D over a wide range of conditions. A distinguishing feature of Model 2 is that the KIR molecules co-cluster with SFKs in the KIR clusters. Live cell imaging of KIR2DL1 in YTS human tumor cell lines with YFP tagged Lck showed the presence of Lck in the KIR microclusters of inhibitory NK synapse lending support to the above hypothesis (29). In Model 2, as the KIR density along with the SFK density in the KIR clusters is decreased with increasing antagonist peptide concentrations, the diffusion time scales for the KIRs and SFKs to meet and enable SFKs to phosphorylate ITIMs is increased leading to lower ITIM phosphorylation and consequently to higher Vav phosphorylation. Thus, in Model 2, the change in the nature of KIR clustering is the major regulator of the suppression of inhibitory signals by antagonist peptides. The behavior holds for a range of parameter values in the model (Figures S5, S6) including variation in the affinities of HLA-C:peptide KIR interactions.

In Model 1, KIRs do not co-cluster with the SFKs, therefore, decreasing the KIR density alone in the spatial clusters with the increasing concentration of antagonist peptides in the peptide mixture does not increase the average distance between the SFKs and KIRs as much as Model 2. As a result, the rate of ITIM phosphorylation is not decreased appreciably with increasing antagonist peptide concentration in Model 1. In addition, the negative feedback in Model 1 owing to the de-phosphorylation of ITIMs by KIR:HLA-C-L7D bound phosphatase SHP1, is decreased as the distance between the KIR molecules increases with increasing antagonist concentrations. Thus, the effect of this reaction becomes weaker with increasing antagonist peptide concentrations. Model 1 is able to reproduce the peptide antagonism behavior if the KIR density in the KIR cluster did not change substantially with increasing antagonist peptide concentration (Figure S4). However, confocal imaging of KIR clusters showed a clear decrease in KIR density in the KIR clusters as the concentration of antagonist peptides is increased (13). Thus, the failure of Model 1 in describing peptide antagonism in KIRs suggested that the co-clustering of KIR and SFKs and breakdown of tight clustering with increase in antagonist concentration are two major mediators of antagonism to KIR inhibition.

Additionally, we have considered a model whereby the loss of the inhibitory signal is related purely to lower levels of KIR engagement by HLA-C molecules expressing the antagonistic peptide (Supplementary Material and Methods). In this analysis we considered the scenario where KIR are limiting. However, this model suggests that KIR engagement in the presence of inhibitory and antagonist peptides is greater than for the inhibitory peptide alone. Additionally we show that the intensity of KIR clusters for the inhibitory:antagonist peptide combination is the same as for the antagonist peptide alone. If our model for antagonism were simply due to lower levels of engagement by antagonist peptide:MHC complexes then we would expect there to be more higher intensity clusters with the inhibitory:antagonist peptide combination than with the antagonist peptide:MHC complexes alone. Overall, therefore our analyses imply that loss of the inhibitory signal is not purely related to the lack of engagement of KIR by the antagonist peptide:MHC complexes.

KIR2DL2 and KIR2DL3 bind a diverse group of HLA-C alleles, most commonly with an asparagine at residue 80. Comparing HLA-C^*^0102 and HLA-C^*^0304 shows that even within this motif-based system single amino acids can have different effects. Thus, for the peptide bound by HLA-C^*^0102 phenylalanine at p7 induces stronger KIR binding than arginine p7, but the reverse is true for HLA-C^*^0304. Additionally, the antagonist peptide for HLA-C^*^0102 has a much slower off-rate from HLA-C than the inhibitory peptide, but this was not true for HLA-C^*^0304. Thus, it appears that stabilizing HLA-C, is enough to disrupt immune synapse formation and subsequent inhibitory signaling. This mechanism was supported by our modeling experiments. The KIR are a rapidly evolving gene family likely driven by the exposure of humans to many different viral infections. Viruses have multiple mechanisms for disrupting MHC class I signaling, but by recruiting the host protein translational machinery they can dramatically affect the peptide content of MHC class within the first few hours following infection (32). Sensing changes in peptide repertoire is a potential mechanism by which KIR-positive NK cells could rapidly recognize a viral infection.

## Ethics Statement

This study was carried out in accordance with the recommendations of name of guidelines, name of committee with written informed consent from all subjects. All subjects gave written informed consent in accordance with the Declaration of Helsinki. The protocol was approved by the NRES (UK) reference: 06/Q1701/120.

## Author Contributions

BM and SK designed the bench experiments. SM and JD designed the *in-silico* models. BM, DW, and SK performed experiments. SM and JD performed computer simulations. BM, SM, JD, and SK analyzed the data and wrote the manuscript.

### Conflict of Interest Statement

The authors declare that the research was conducted in the absence of any commercial or financial relationships that could be construed as a potential conflict of interest.
