# Corrigendum: Spatial Clustering of Receptors and Signaling Molecules Regulates NK Cell Response to Peptide Repertoire Changes

**DOI:** 10.3389/fimmu.2019.02370

**Published:** 2019-10-15

**Authors:** Berenice Mbiribindi, Sayak Mukherjee, Dannielle Wellington, Jayajit Das, Salim I. Khakoo

**Affiliations:** ^1^Department of Clinical and Experimental Sciences, Faculty of Medicine, University of Southampton, Southampton, United Kingdom; ^2^Institute of Bioinformatics and Applied Biotechnology, Bangalore, India; ^3^Department of Cancer Sciences, Faculty of Medicine, University of Southampton, Southampton, United Kingdom; ^4^Battelle Center for Mathematical Medicine, The Research Institute at the Nationwide Children's Hospital, Columbus, OH, United States; ^5^Department of Pediatrics, Wexner College of Medicine, The Ohio State University, Columbus, OH, United States; ^6^Biophysics Program, The Ohio State University, Columbus, OH, United States

**Keywords:** NK cells, peptide antagonism, KIR, HLA-C, signaling, modeling

In the original article, there was a mistake in the **Supplementary Figures S1–S6** as published. None of the images for the figures appeared in the final publication. The missing images have been added to the original article's supplementary file.

The authors apologize for this error and state that this does not change the scientific conclusions of the article in any way. The original article has been updated.

