# Supplementary material for: Spatial Clustering of Receptors and Signaling Molecules Regulates NK Cell Response to Peptide Repertoire Changes
Source: Front Immunol. 2019 Apr 5;10:605. doi: 10.3389/fimmu.2019.00605 (PMC6460049; doi:10.3389/fimmu.2019.00605)
Supplement: Supplementary file 1 [file Data_Sheet_1.pdf]

Spatial clustering of receptors and signaling molecules regulates NK cell response to peptide repertoire changes

Berenice Mbiribindi[1], Sayak Mukherjee[2], Dannielle Wellington[3], Jayajit Das[4,5,6*] and Salim I. Khakoo[1*]

[1]Department of Clinical and Experimental Sciences, Faculty of Medicine, University of Southampton, SO16 6YD Southampton, UK
[2]Institute of Bioinformatics and Applied Biotechnology, Bangalore, India 560100
[3]Department of Cancer Sciences, Faculty of Medicine, University of Southampton, SO16 6YD Southampton, UK
[4]Battelle Center for Mathematical Medicine, The Research Institute at the Nationwide Children's Hospital, Columbus, OH 43205, USA
Department of [5]Pediatrics, Wexner College of Medicine, and [6]Biophysics Program, the Ohio State University, Columbus, OH 43210, USA

***Corresponding authors:**

Prof Salim Khakoo
Faculty of Medicine
Southampton General Hospital
University of Southampton
Mailpoint 811, Level E South Academic Block, Tremona Road
Southampton SO16 6YD, UK
Tel.: +44 023 8021 4004/5099
e-mail: s.i.khakoo@soton.ac.uk
and

Dr. Jayajit Das
Battelle Center for Mathematical Medicine,
The Research Insitute at the Nationwide Childrens Hospital and the Department of Pediatrics, The Ohio State University,
700 Childrens Drive,
Columbus, OH 43205 USA
Tel: +1 614 355 5632
Jayajit.Das@nationwidechildrens.org

Supplemental informations

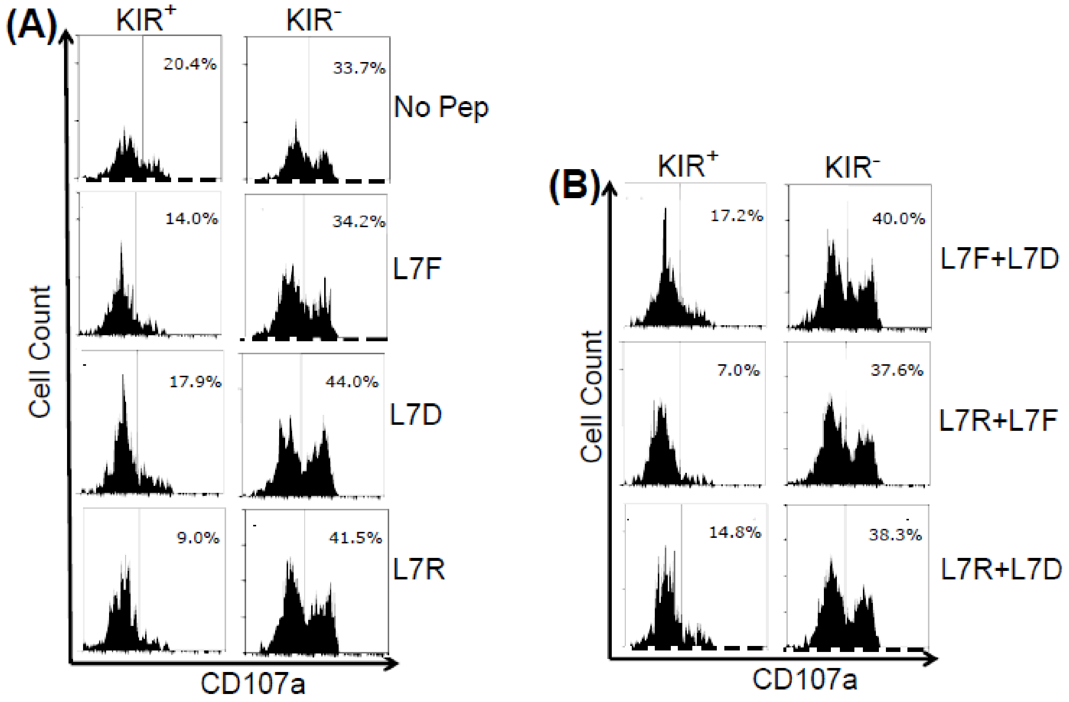

Figure S1. Flow cytometry plots of NK cell degranulation assay using p7 peptides variants. 721.221C*0304-ICP47 cells were loaded with the indicated peptides then used as targets for IL-15 activated NK cells. CD107a expression of KIR2DL3+ or KIR2DL3- NK cells after co-culture with target cells loaded with the indicated single peptides at a concetration of 12.5μM (A) or combination of peptides at a concentration of 12.5μM (B).

<u>Details of simulation—Methods and Material section</u>

We simulated membrane proximal signaling in Natural Killer (NK) cells using the standard spatial stochastic Gillespie simulation (Lis et al. 2009; Gillespie 1976). We subdivided the entire simulation volume into 10^4 small cubes of length *l* = 20 nm (**Figure S2**). The length *l* was chosen to be smaller than the root mean square displacement of a typical membrane bound molecule such that $l < \sqrt{\langle \Delta x^2 \rangle} = \sqrt{4D_{mem} t}$, where D_{mem} is the diffusion constant of membrane bound macromolecules, taken to be $D_{mem} = 10^{-2}$ μm^2 s^{-1}, and *t* is the timescale of a typical fast reaction, which was set to be 0.1 s^{-1} in our simulation. This choice of *l* ensured that reactants were well mixed within a cube of length *l* and consequently the reactions inside the cube could be modeled using mass action kinetics. Diffusion itself was implemented as a hoping reaction, whereby a molecule inside a sub-volume hopped onto one of its four nearest neighboring sub-volumes with a

hoping rate $h = D / l^2$. Those molecules that were not bound to the plasma membrane possessed a larger diffusion constant of 10 μm^2 s^{-1} and consequently diffused three orders of magnitude faster to their membrane bound counterpart. Reactions within a sub-volume were implemented using mass action kinetics where propensity of a reaction is given by the product of the concentrations of the reactants. The list of possible reactions inside a sub-volume and their respective rate constants are shown in **Table S1-S2**. The number of molecules used in our simulation is given in **Table S3**. We have implemented a strict kinetic proof reading scheme where disengagement of the ligands resulted in immediate and full breakup of the receptor-ligand complex. The K_D (=k_{on}/k_{off}) values for the binding/unbinding of the inhibitory (L7R) and the antagonist (L7D) peptides were estimated to be 0.8 μM and 4.3 μM, respectively, from the ligand binding experiments in **Figure 1C** in the main text. We estimated k_{on} and k_{off} values (**Table S1-S2**) for these ligands that are consistent with the above K_D values. The variation of the k_{off} values show that the qualitative behavior of our modeling results are not sensitive to the specific values used in the model within a range (**Figure S6**).

<u>Initial distribution of molecules</u>

<u>Model 1 or negative feedback model:</u> Activating receptors and their cognate ligands were distributed homogeneously across the simulation box in the beginning of our simulation. The inhibitory KIR receptors are known to form micro-clusters after binding to their cognate ligands (Treanor et al. 2006). Here, we assumed that both KIRs and their ligands reside in a small preformed cluster in the middle of the simulation box (**Figure S2**). Previously, it was reported that KIRs bound to strong inhibitory peptides form tight KIR clusters whereas addition of "antagonist" peptides result it disruption of tight cluster formation (Borhis et al. 2013). We incorporated this fact by making the size of preformed cluster (ξ) grow hyperbolically with "antagonist concentration" as shown in Eqn S1. A fixed number of KIR molecules is distributed homogenously within the cluster, therefore, as the cluster size increases the density of the KIR moelcules within the cluster decreases.

$$\xi = a + \frac{b[L_2]}{K_R + [L_2]} \qquad \text{S1}$$

where a is the cluster size in absence of any "antagonist", $[L_2]$ is the concentration of "antagonist" peptide and K_R is a repression co-efficient that one can choose a value for. When antagonist concentration $[L_2] >> K_R$, the cluster size reaches the maximum value of $a + b$. The magnitude of K_R determines how quickly ξ reaches the saturation value as the number of antagonist peptide is increased. Therefore, by changing K_R we have explored the effect that different rates of increase in the cluster sizes as $[L_2]$ is increased in the simulation. As Eqn S1 does not depend on the concentration of strong inhibitory peptide, we have tacitly assumed that the cluster size does not change with a change inhibitory peptide concentration. We used a = 0.2 μm and b = 1.8 μm and a

K_R value of 5 μM for **Figure 7** in the main text. The rest of the proteins such as the Src family kinase, Lck, Syk family kinase Zap70 and phosphatase SHP-1 are distributed homogenously at the beginning of our simulation. Vav-1 is a downstream target of both Zap70 and SHP-1 and also an activation marker for CD107 induction in NK cell (Borhis et al. 2013). Vav-1 was distributed uniformly across the simulation box.

<u>Model 2 or Cluster dynamics model</u>

Distribution of proteins in Model 2 is almost similar to Model 1 with just a few notable differences. Here, like before, KIR and KIR binding peptides resided inside a preformed micro-cluster at the beginning of our simulation. But we also co-localized almost half of the available pool of Lck molecules inside the micro-cluster with the inhibitory receptors. It is worth mentioning that co-localization of Lck inside the inhibitory cluster is an essential ingredient of this model. As cluster size was increased, the effective on rate for phosphorylation of ITIM by Lck decreased. Previously, using resonance scanning confocal microscopy, Treanor et al. demonstrated that Lck indeed co-localizes with KIR2DL1 at the immunological synapse in YTS/KIR2DL1 cells transfected with Lck conjugated with monomeric YFP in presence of 221/Cw6 target cells (Treanor et al. 2006) lending suport for this crucial assumption. As for the rest of Lck, we distributed them uniformly outside the micro-cluster. Lck molecules that were initially inside (or outside) the microcluster were kept inside (or outside) during rest of the simulation (**Figure S2B**). Like Model 1, we have used a = 0.2 μm, b = 1.8 μm and K_R = 5 μM for Model2.

A simple mathematical model demonstrates that more KIRs are engaged for mixed peptide case to single peptide case.

We compared two models of KIR antagonism where antagonism to KIR inhibition was explained either by a specific negative feedback induced by ITIM bound SHP-1 (Model 1) or by lower co-aggregation of SFKs with KIRs in the presence of the antagonists(Model 2). A question can however be raised if a more simplistic mechanism where a lower number of inhibitory KIRs are engaged when NK cells are stimulated with a mixture of L7R(inhibitory) and L7D(antagonist) ligands as compared to when NK cells stimulated with the same concentration of L7R alone can be proposed to explain the KIR antagonism. The following simple example is constructed to provide a precise description of the above proposal. Consider [R] number of inhibitory KIRs interacting with [L_1] number of inhibitory ligands (single peptide stimulation) or [L_1] number of inhibitory+[L_2] number of antagonist ligands (mixed peptide stimulation). The above mechanism states that the number of KIRs engaged for the mixed peptide simulation will be less compared to the single peptide stimulation. Therefore, the strength of the inhibitory signal which is likely to be proportional to the number of engaged inhibitory KIRs will be lower for the mixed stimulation compared to the single peptide stimulation providing a simple mechanism regarding how

antagonist ligands suppress inhibitory signals. Below we show that this simple minded picture does not hold true in our simple example.

Consider the following binding unbinding reactions for inhibitory KIRs with inhibitory (L_1) and antagonist ligands(L_2):

Single peptide stimulation	Mixed peptide stimulation
$R + L_1 \underset{(k_{off})_1}{\overset{(k_{on})_1}{\rightleftarrows}} RL_1$	$R + L_1 \underset{(k_{off})_1}{\overset{(k_{on})_1}{\rightleftarrows}} RL_1$<br>$R + L_2 \underset{(k_{off})_2}{\overset{(k_{on})_2}{\rightleftarrows}} RL_2$

For the single peptide case, we have the KIRs (R) binding to the MHC-inhibitory peptide L7R complex (L_1) to form KIR-L7R-MHC (RL_1) with a dissociation constant $k_D^{(I)}$ =$(k_{off})_1/ (k_{on})_1$. For the mixed peptide case, we have KIR binding to the MHC-antagonist peptide L7D complex (L_2) to form KIR-L7D-MHC (RL_2) with a dissociation constant of $k_D^{(II)}$ =$(k_{off})_2/ (k_{on})_2$ in addition to the reactions mediated by the inhibitory peptide. First we analyse the case for single peptide stimulation. In the steady state, the number (x^{sp}) of KIRs bound to their cognate ligands for the single peptide case is given by

$$k_D^{(I)} x^{sp} = [R][L_1] \qquad \text{S2}$$

where [] represents the number of molecules in the cell. For simplifying the algebraic equations we denote, $[RL_1]=x$, and, $[RL_2]=y$. Using the conservation equations $[R] + [x] = R_0$ and $[L_1]+[x]=L_1^0$, R_0 and L_1^0 are the initial numbers of the KIRs and MHC-L7R complex respectively, we can write Eq. [S2] as

$$k_D^{(I)} x^{sp} = [R_0 - x^{sp}][L_1^0 - x^{sp}]. \qquad \text{S3}$$

Now we consider the case where the number of KIRs is limiting, i.e., $[R_0] \ll [L_1^0]$ and $[R_0] \ll [L_2^0]$. This is because when the KIRs are present in excess compared to the numbers of inhibitory and the antagonist ligands, each of the ligand type will not have difficulty in finding partnering KIRs, thus the mixed stimulation will contain a higher number of bound KIRs compared to that for the single peptide stimulation. The competition between the inhibitory and the antagonist ligands will become dominant when the number of KIRs are present in much smaller numbers than the ligand numbers.

In the case of limiting KIR numbers, $x/R_0 <1$, $x/L_1 < 1$ and $x/L_1 << x/R_0$ (as R_0 is limited). Ignoring term of $O((x/ L_1^0) .(x/R_0))$, we can write Eq. (S3) as

$$x^{sp} = \frac{R_0 L_1^0}{R_0 + L_1^0 + k_D^{(I)}}$$

Similarly for the mixed peptide stimulation case, one can easily show that $k_D^{(I)} x^{mp} = [R_0 - x^{mp} - y^{mp}][L_1^0 - x^{mp}]$, where x^{mp} and y^{mp} are steady state numbers of KIR-L7R-MHC and KIR-L7D-MHC respectively. The superscript *mp* stands for mixed peptide. Again, ignoring terms of $O((x/L_1^0).(x/R_0))$ & $O((x/L_1^0).(y/R_0))$, we get

$$\begin{aligned} x^{mp} &= \frac{R_0 L_1^0}{R_0 + L_1^0 + k_D^{(I)}} - \frac{L_1^0 y^{mp}}{R_0 + L_1^0 + k_D^{(I)}} \\ &= x^{sp} - \frac{L_1^0 y^{mp}}{R_0 + L_1^0 + k_D^{(I)}} \end{aligned}$$

Therefore the total number of KIR engaged in the mixed peptide case ($x^{mp} + y^{mp}$) is

$$x^{mp} + y^{mp} = x^{sp} + \left(1 - \frac{L_1^0}{R_0 + L_1^0 + k_D^{(I)}}\right) y^{mp} \qquad \text{S4}$$

Since the term inside the parenthesis in Eq. (S4) is always greater than zero, $x^{mp} + y^{mp} > x^{sp}$. Therefore, more KIRs will be bound to the ligands in the mixed peptide compared to the single peptide case even when the number of KIRs is limited ($R_0 << L_1^0$ or $R_0 << L_2^0$). The the premise of the simple mechanism, i.e., the number of KIRs engaged for the mixed peptide simulation will be less compared to the single peptide stimulation, does not hold good in the simple example.

Table S1. Reactions and reaction rates used in Model 1

Reactions	k_{on} ($\mu M^{-1}s^{-1}$)	k_{off} (s^{-1})	k_{cat} (s^{-1})
$AR + L_{act} \rightleftarrows AR_{bound}$<br>Binding unbinding of activating receptor (*AR*) to its cognate ligand (L_{act})	0.125	0.1	
$AR_{bound} + Lck \rightleftarrows AR_{bound} - Lck \rightarrow AR^{*}_{bound} + Lck$<br>Upon ligation ($AR_{bound}$), Lck binds and unbinds AR_{bound} and phosphorylates ITAMs in the cytoplasmic tail of *AR* (AR^{*}_{bound})	0.025	1.0	0.1
$AR^{*}_{bound} \rightarrow AR_{bound}$<br>ITAMs can be de-phosphorylated by uncharacterized phosphatases			0.05
$AR^{*}_{bound} + Zap70 \rightleftarrows AR^{*}_{bound} - Zap70$<br>Syk family kinase, Zap-70, binds to the phos-ITAMs	0.1	1.0	
$AR^{*}_{bound} - Zap70 + Vav \rightleftarrows AR^{*}_{bound} - Zap70 - Vav$<br>$\rightarrow Vav^{*} + AR^{*}_{bound} - Zap70$<br>Receptor bound Zap-70 then phosphorylates its downstream target Vav1 (Vav^{*})	0.025	0.1	1.5
$H - L_1 \rightarrow H + L_1$<br>Unbinding of strong inhibitory peptide (L_1) from the surface of HLA-C molecules (*H*)			10^{-4}
$IR + H - L_1 \rightleftarrows IR_{b_1}$<br>Inhibitory receptor KIR (*IR*) binds strong inhibitory peptide loaded on the surface of HLA-C (H-L_1)	0.1	0.1	
$IR_{b_1} + Lck \rightleftarrows IR_{b_1} - Lck \rightarrow IR^{*}_{b_1} + Lck$<br>KIR bound to strong inhibitory peptide (IR_{b1}) binds Lck, which phosphorylates ITIMs on KIRs cytoplasmic tail	0.025	1.0	0.1

$H-L_2 \rightarrow H+L_2$<br>Unbinding of antagonist peptide (L_2) from the surface of HLA-C molecules (*H*)			10^{-4}
$IR+H-L_2 \rightleftarrows IR_{b_2}$<br>*IR* binds antagonist peptide loaded on the surface of HLA-C (H-L_2)	0.1	0.4	
$IR_{b_2}+Lck \rightleftarrows IR_{b_2}-Lck \rightarrow IR^*_{b_2}+Lck$<br>KIR bound antagonist peptide (IR_{b2}) binds Lck, which phosphorylates ITIMs on KIRs cytoplasmic tail	0.025	1.0	0.1
$IR^*_{b_1/b_2}+SHP \rightleftarrows IR^*_{b_1/b_2}-SHP$<br>Phosphorylated ITIMs in the cytoplasmic tail recruits SHP-1 (*SHP*)	0.1	1.0	
$IR^*_{b_1/b_2} \rightarrow IR_{b_1/b_2}$<br>Phosphorylated ITIMs can get de-phosphorylated by uncharacterized phosphatases			0.05
$IR^*_{b_2}-SHP+IR^*_{b_1/b_2} \rightarrow IR^*_{b_2}-SHP+IR_{b_1/b_2}$<br>SHP bound to IR^*_{b2} de-phosphorylates phosphorylated ITIMs of neighboring *IR*s	2.5		
$IR^*_{b_1/b_2}-SHP+Vav^* \rightleftarrows IR^*_{b_1/b_2}-SHP-Vav^*$<br>$\rightarrow Vav + IR^*_{b_1/b_2}-SHP$<br>KIR bound SHP then de-phosphorylates its downstream target Vav^*	0.125	0.1	1.5

Table S2. Reactions and reaction rates used in Model 2

Reactions	K_{on} ($\mu M^{-1}s^{-1}$)	K_{off} (s^{-1})	K_{cat} (s^{-1})
$AR + L_{act} \rightleftarrows AR_{bound}$<br>Binding unbinding of activating receptor (*AR*) to its cognate ligand (L_{act})	0.125	0.1	
$AR_{bound} + Lck \rightleftarrows AR_{bound} - Lck \rightarrow AR^{*}_{bound} + Lck$<br>Upon ligation ($AR_{bound}$), Lck binds and unbinds AR_{bound} and phosphorylates ITAMs in the cytoplasmic tail of *AR* (AR^{*}_{bound})	0.025	1.0	0.1
$AR^{*}_{bound} \rightarrow AR_{bound}$<br>ITAMs can be de-phosphorylated by uncharacterized phosphatases			0.0<br>5
$AR^{*}_{bound} + Zap70 \rightleftarrows AR^{*}_{bound} - Zap70$<br>Syk family kinase, Zap-70, binds to the phos-ITAMs	0.1	1.0	
$AR^{*}_{bound} - Zap70 + Vav \rightleftarrows AR^{*}_{bound} - Zap70 - Vav$<br>$\rightarrow Vav^{*} + AR^{*}_{bound} - Zap70$<br>Receptor bound Zap-70 then phosphorylates its downstream target Vav1 (Vav^{*})	0.025	0.1	0.5
$H - L_1 \rightarrow H + L_1$<br>Unbinding of strong inhibitory peptide (L_1) from the surface of HLA-C molecules (*H*)			10^{-4}
$IR + H - L_1 \rightleftarrows IR_{b_1}$<br>Inhibitory receptor KIR (*IR*) binds strong inhibitory peptide loaded on the surface of HLA-C (H-L_1)	0.1	0.1	
$IR_{b_1} + Lck \rightleftarrows IR_{b_1} - Lck \rightarrow IR^{*}_{b_1} + Lck$<br>KIR bound to strong inhibitory peptide (IR_{b1}) binds Lck, which phosphorylates ITIMs on KIRs cytoplasmic tail	0.0125	5.0	0.1

$H-L_2 \rightarrow H+L_2$<br>Unbinding of antagonist peptide (*L_2*) from the surface of HLA-C molecules (*H*)			10^{-4}
$IR+H-L_2 \rightleftarrows IR_{b_2}$<br>*IR* binds antagonist peptide loaded on the surface of HLA-C (*H-L_2*)	0.1	0.4	
$IR_{b_2}+Lck \rightleftarrows IR_{b_2}-Lck \rightarrow IR^*_{b_2}+Lck$<br>KIR bound antagonist peptide (*IR_{b2}*) binds Lck, which phosphorylates ITIMs on KIRs cytoplasmic tail	0.0125	5.0	0.1
$IR^*_{b_1/b_2}+SHP \rightleftarrows IR^*_{b_1/b_2}-SHP$<br>Phosphorylated ITIMs in the cytoplasmic tail recruits SHP-1 (*SHP*)	0.125	1.0	
$IR^*_{b_1/b_2} \rightarrow IR_{b_1/b_2}$<br>Phosphorylated ITIMs can get de-phosphorylated by uncharacterized phosphatases			0.1
$IR^*_{b_1/b_2}-SHP+Vav^* \rightleftarrows IR^*_{b_1/b_2}-SHP-Vav^*$<br>$\rightarrow Vav+IR^*_{b_1/b_2}-SHP$<br>KIR bound SHP then de-phosphorylates its downstream target Vav^*	0.125	0.1	1.5

Table S3. Concentrations used

Molecules	Concentration (μM)
AR	80
IR	20
L_{act}	25
L_1/L_2	0-12.5
Lck	90
Zap-70	10
SHP	10
Vav	12.5

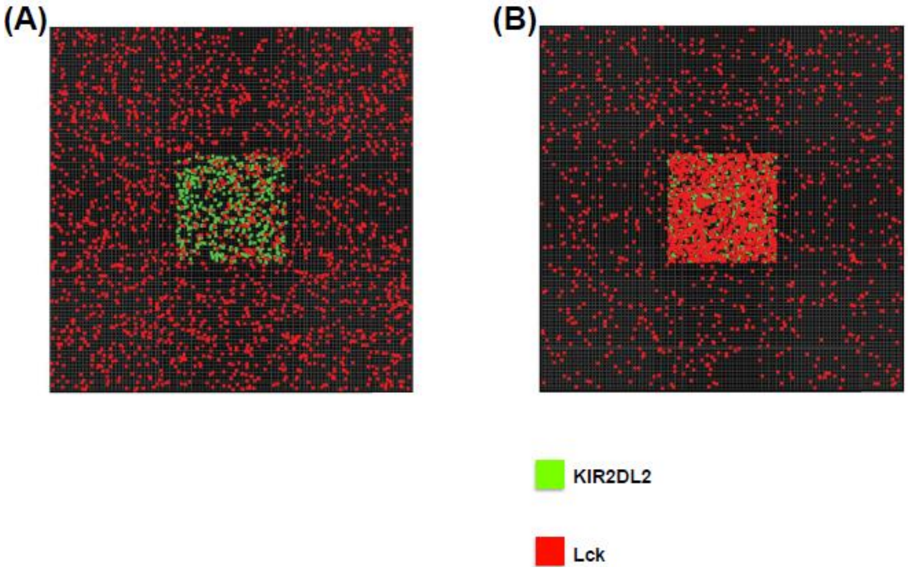

Figure S2. Distribution of KIR2DL2 inhibitory receptors and SFKs in Model 1 & Model 2. (A) In Model 1**,** KIRs (shown in green) form a microcluster of size 0.2 to 1.8 μm in the middle of the simulation box. Lck molecules (shown in red) do not co-cluster with the KIRs, instead they are distributed homogenously across the simulation box. (B) As before, KIRs form a cluster of same size as in (A) in the middle of the simulation box in Model 2. But almost half of the population of available Lck also co-clusters with the KIRs leading to an enhanced concentration of Lcks inside the KIR cluster. The remaining half is distributed homogenously in the rest of the simulation box.

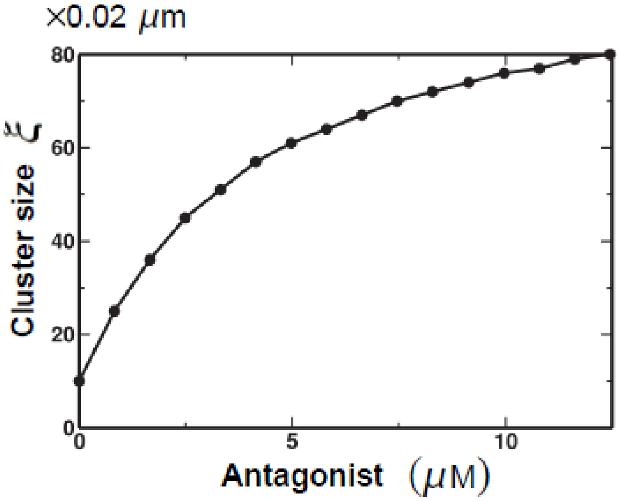

Figure S3. Cluster size increases hyperbolically as a function of antagonist concentration. The black curve shows the variation of cluster size ξ as a function of antagonist concentration. A fixed number of KIR molecules is distributed homogeneously within the cluster. Thus, as the cluster size ξ increases, the density of KIR molecules in the cluster decreases.

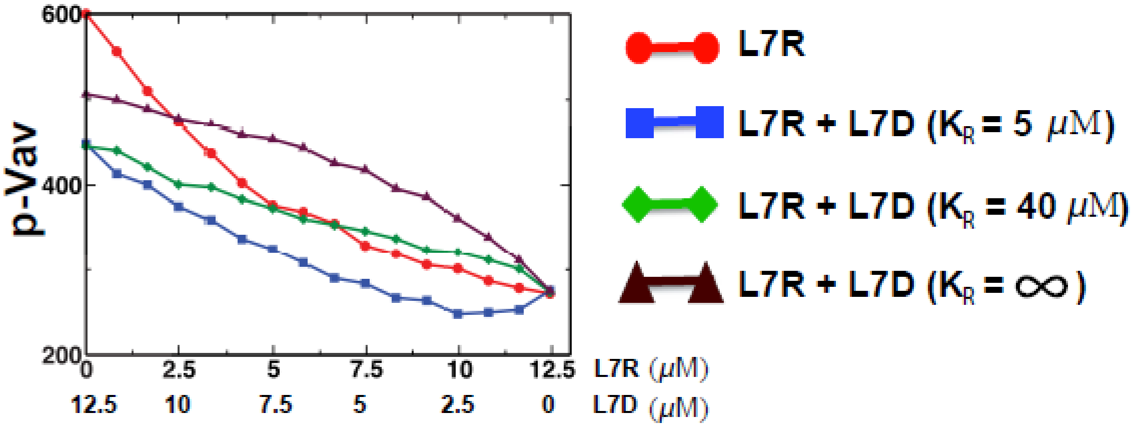

Figure S4. Model 1 can account for antagonism to inhibition when cluster size does not change appreciably with the increase in antagonist concentration. In the figure above, the red curve shows the change in p-Vav numbers as inhibitory peptide L7R concentration is increased from 0 to 12.5 μM. For this case, he cluster size is held fixed at ξ = 0.2 μm. All the other curves (blue, green and maroon) show the variation in p-Vav numbers as L7D antagonist peptide concentration is increased from 0 to 12.5 μM but for different rates of change of cluster size ξ. For the blue curve the cluster size changes from 0.2 μm to 1.6 μm (K_R = 5 μM) as the concentration of L7D is increased. For the green curve the cluster size change from 0.2 μm to 0.6 μm (K_R = 40 μM). For the maroon curve the cluster size is 0.2 μm and does not change at all ($K_R = \infty$). In all these plots the variation in L7D concentration is done in such a way that the sum of L7R and L7D is always 12.5 μM.

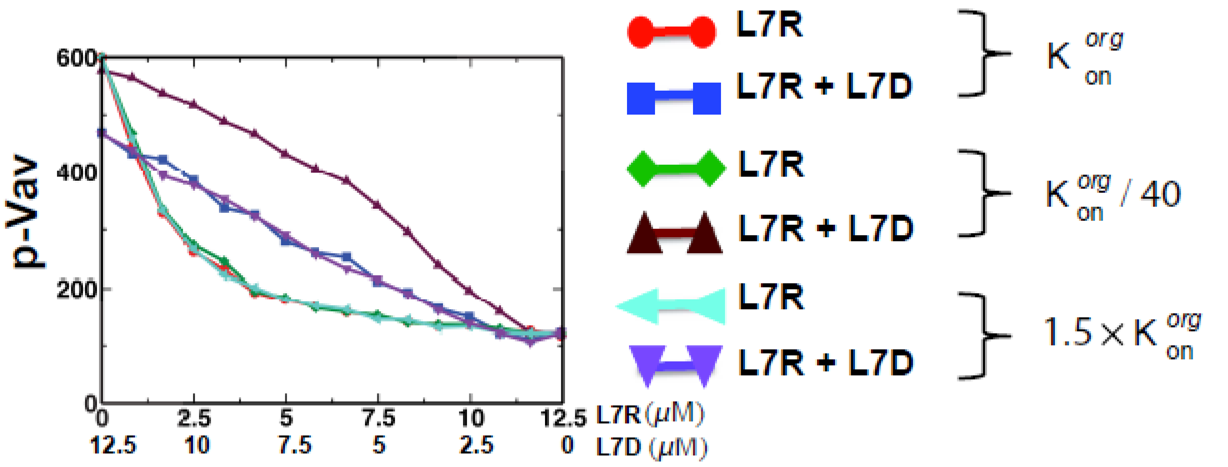

Figure S5. Reducing the binding rate of peptide to KIR leads to an increase in antagonism to inhibition. When k_{on} is lowered 40 times (green and brown plots) the antagonism to inhibition is

magnified, whereas increasing k_{on} by 1.5 times (cyan and violet plots) or more do not lower antagonism substantially.

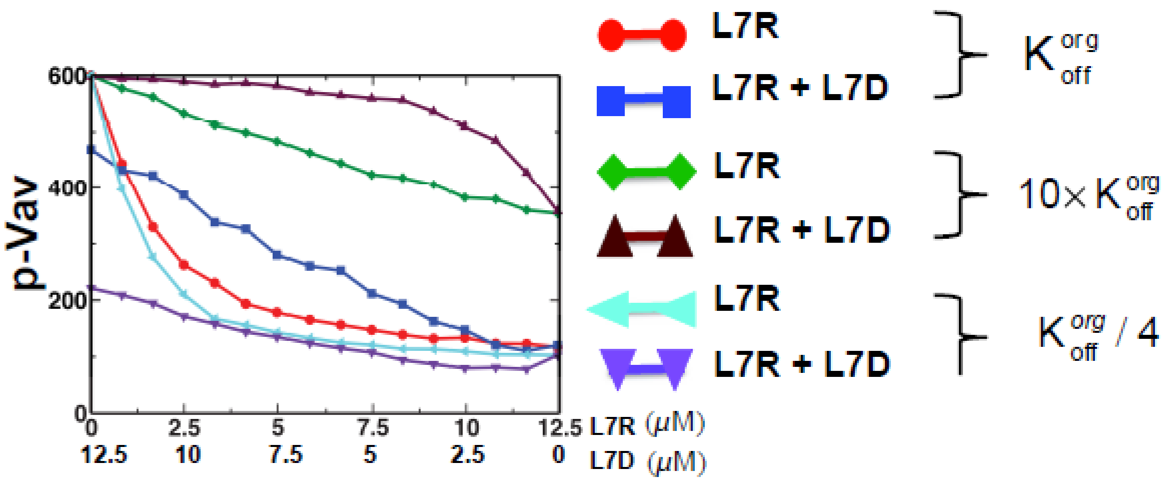

Figure S6. Increase in the unbinding rate of peptide from KIR leads to an increase in antagonism to inhibition. When k_{off} is increased 10 times (green and brown plots) the antagonism to inhibition is magnified, whereas decreasing k_{off} by 4 times (cyan and violet plots) or more leads to loss of antagonism to inhibition. This loss of antagonism can be recovered by if the number of Lck co-localized inside the micro-cluster is concurrently lowered
